# APOBEC3A drives ovarian cancer metastasis by altering epithelial-mesenchymal transition

**DOI:** 10.1172/jci.insight.186409

**Published:** 2025-03-10

**Authors:** Jessica M. Devenport, Thi Tran, Brooke R. Harris, Dylan Fingerman, Rachel A. DeWeerd, Lojain H. Elkhidir, Danielle LaVigne, Katherine Fuh, Lulu Sun, Jeffrey J. Bednarski, Ronny Drapkin, Mary M. Mullen, Abby M. Green

**Affiliations:** 1 Department of Pediatrics,; 2 Cancer Biology Graduate Program, and; 3 Molecular Genetics and Genomics Graduate Program, Washington University School of Medicine, St. Louis, Missouri, USA.; 4 Department of Obstetrics, Gynecology, and Reproductive Sciences, UCSF, San Francisco, California, USA.; 5 Division of Anatomic and Molecular Pathology, Department of Pathology and Immunology, Washington University School of Medicine, St. Louis, Missouri, USA.; 6 Penn Ovarian Cancer Research Center, Department of Obstetrics and Gynecology, and; 7 Basser Center for BRCA, Abramson Cancer Center, University of Pennsylvania Perelman School of Medicine, Philadelphia, Pennsylvania, USA.; 8 Division of Gynecologic Oncology, Department of Obstetrics and Gynecology, Siteman Cancer Center, and; 9 Center for Genome Integrity, Siteman Cancer Center, Washington University School of Medicine, St. Louis, Missouri, USA.

**Keywords:** Cell biology, Oncology, Cancer, Cell migration/adhesion, DNA repair

## Abstract

High-grade serous ovarian cancer (HGSOC) is the most prevalent and aggressive histological subtype of ovarian cancer and often presents with metastatic disease. The drivers of metastasis in HGSOC remain enigmatic. APOBEC3A (A3A), an enzyme that generates mutations across various cancers, has been proposed as a mediator of tumor heterogeneity and disease progression. However, the role of A3A in HGSOC has not been explored. We observed an association between high levels of APOBEC3-mediated mutagenesis and poor overall survival in primary HGSOC. We experimentally addressed this correlation by modeling A3A expression in HGSOC, and this resulted in increased metastatic behavior of HGSOC cells in culture and distant metastatic spread in vivo, which was dependent on catalytic activity of A3A. A3A activity in both primary and cultured HGSOC cells yielded consistent alterations in expression of epithelial-mesenchymal transition (EMT) genes resulting in hybrid EMT and mesenchymal signatures, providing a mechanism for their increased metastatic potential. Inhibition of key EMT factors TWIST1 and IL-6 resulted in mitigation of A3A-dependent metastatic phenotypes. Our findings define the prevalence of A3A mutagenesis in HGSOC and implicate A3A as a driver of HGSOC metastasis via EMT, underscoring its clinical relevance as a potential prognostic biomarker. Our study lays the groundwork for the development of targeted therapies aimed at mitigating the deleterious effect of A3A-driven EMT in HGSOC.

## Abstract 2

We found that APOBEC3A, an innate immune enzyme involved in virus restriction, acts abnormally in ovarian cancer cells to promote metastasis.

## Introduction

Ovarian cancer is the fifth most prevalent cancer among women and is the leading cause of death from gynecologic malignancies worldwide (1). High-grade serous ovarian cancer (HGSOC) represents the most frequent and aggressive histological subtype of ovarian cancer, accounting for approximately 70%–80% of ovarian cancer-related deaths (2). HGSOC is often diagnosed at a late stage and exhibits extensive intra- and intertumor heterogeneity, resulting in significant challenges in clinical management (3). Intratumor heterogeneity in HGSOC manifests as diverse subclones with distinct genomic alterations and gene expression profiles within individual tumors. This clonal diversity is associated with treatment resistance and disease progression (2, 4, 5). Despite aggressive therapeutic interventions, most patients with HGSOC experience relapse and diminished survival, highlighting the critical need for a deeper understanding of the molecular drivers of HGSOC disease progression.

Cancer genome sequencing has established that APOBEC3 (apolipoprotein B mRNA editing enzyme, catalytic polypeptide-like 3) enzymes play a significant role in promoting widespread mutagenesis across various tumor types (4, 6–12). The APOBEC3 family of cytidine deaminases (APOBEC3A–APOBEC3H [A3A–A3H]) induce mutagenesis through deamination of cytidine to uracil in single-stranded DNA (ssDNA). APOBEC3 enzymes normally act as virus restriction factors (13). However, aberrant deaminase activity by APOBEC3 enzymes results in damage and mutagenesis to the cellular genome (14–16). Mutations resulting from the enzymatic activity of 2 APOBEC3 family members, A3A and A3B, leave distinct mutational patterns, defined as single-base substitution (SBS) signatures 2 and 13 in the Catalogue of Somatic Mutations in Cancer (COSMIC) database (17). Analyses of tumor genomes have identified APOBEC3 mutagenesis in various cancer types, including breast, bladder, and lung cancer, where is it associated with the accumulation of somatic mutations and the development of subclonal diversity (6, 8, 9, 11, 18–20). Moreover, APOBEC3-mediated mutagenesis has been linked to tumor progression, metastasis, and therapy resistance in breast and lung cancer (19, 21–23).

Detection of APOBEC3 mutational signatures is well established in clear-cell ovarian carcinoma (24–28); however, prior studies have demonstrated a perplexing range in the contribution of APOBEC3 mutagenesis to overall tumor mutation burden in HGSOC. In a study of more than 100 patients with HGSOC, APOBEC3 mutagenesis was found to contribute to approximately 3% of the overall mutational burden in tumor genomes (28). A smaller study assessing the mutational profiles of various gynecologic cancers found that APOBEC3 mutagenesis contributed to a vast range of 0%–70% of the overall mutational burden in 4 patients with HGSOC (25). Thus, the frequency of APOBEC3 mutagenesis in HGSOC remains unclear.

Activity of both A3A and A3B contribute to tumor mutational burden, although several studies have identified A3A as the predominant driver of SBS2 and SBS13 in tumor genomes (8, 29, 30). A germline deletion of *A3B*, which causes *A3A* transcript terminating in the *A3B* 3′UTR, results in increased A3A mutagenesis (31, 32). Epidemiologic studies have demonstrated that this polymorphism is associated with a higher risk of developing tumors, including ovarian cancer (33, 34). The *A3B* deletion polymorphism is also associated with increased APOBEC3 mutational signatures, indicating elevated A3A activity in tumors (32, 35). However, the contribution of A3A activity to genomic heterogeneity and the biological consequences for HGSOC disease progression are unknown.

In addition to genomic heterogeneity, metastatic progression of HGSOC is driven by epithelial-mesenchymal transition (EMT), a dynamic biological process through which epithelial cells undergo a series of molecular changes that shift the cells toward a mesenchymal phenotype (36). During EMT, cells lose epithelial characteristics, such as cell-cell adhesion and apical-basal polarity, and gain mesenchymal traits such as motility, invasiveness, and resistance to apoptosis (36). EMT is vital during embryonic development, wound healing, and tissue generation but can be hijacked by cancer cells, facilitating metastasis and disease progression (37, 38). In HGSOC, EMT plasticity generates phenotypically diverse cancer cell populations that exhibit epithelial and mesenchymal characteristics simultaneously, often termed hybrid EMT (hEMT) (39). In a large-scale analysis of EMT phenotypes in cancer, an enrichment of the APOBEC3-mediated SBS2 and SBS13 was observed in tumors exhibiting hEMT expression profiles (38).

In this study, we examined the prevalence of APOBEC3 mutational signatures in HGSOC genomes and investigated the consequences of A3A mutagenesis on patient outcomes. Through genome sequencing of HGSOC, we found an enrichment of A3A mutagenesis in metastatic tumors. Elevated levels of A3A mutagenesis significantly correlated with reduced patient survival. By modeling expression of A3A in HGSOC we demonstrated that A3A activity promotes prometastatic phenotypes in cultured cells and distant metastatic spread in murine models. Furthermore, we found that A3A activity led to expression of hEMT and mesenchymal genes in HGSOC cells and metastatic patient tumors, providing a mechanistic explanation for accelerated metastasis. Concordant with this, we identified elevated expression and secretion of IL-6 in HGSOC cells exposed to A3A activity. We found that EMT-associated phenotypes in HGSOC cells exposed to A3A was dependent on IL-6, as evidenced by a reversal of EMT-associated phenotypes upon IL-6 blockade. Our study demonstrates the effect of A3A activity on HGSOC progression via EMT, which may be applicable to other tumors, and identifies potential therapeutic vulnerabilities in tumors with high A3A activity.

## Results

### APOBEC3 mutagenesis is enriched in metastatic high-grade serious ovarian cancer.

**Figure 1:**
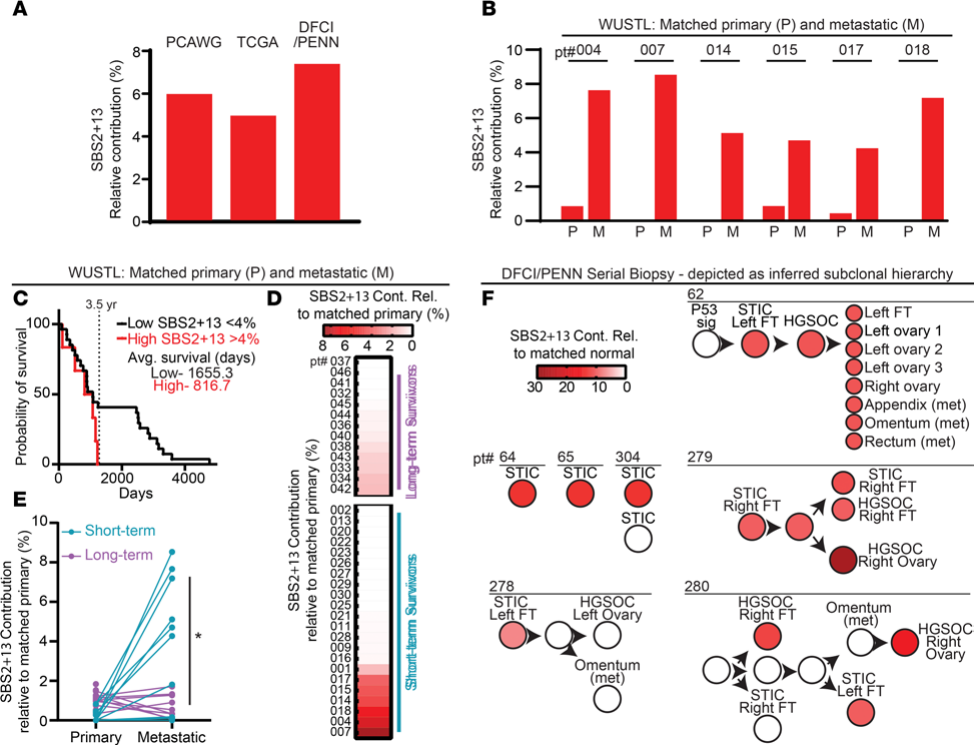
APOBEC3 activity correlates with poor survival in patients with HGSOC. (**A**) Whole genome sequencing was assessed to determine the mutational processes occurring within tumor genomes from PCAWG Ovarian Cancer (PCAWG), TCGA Ovarian Cancer (TCGA), and Dana Farber Cancer Institute/University of Pennsylvania (DFCI/PENN) patient cohorts. Relative contribution of APOBEC3 mutational signatures (SBS2 and SBS13) is shown as a fraction of total mutational burden (PCAWG, 5.9%; TCGA, 4.9%; DFCI/PENN, 7.3%). (**B**–**E**) Whole exome sequencing from a patient cohort at Washington University in St. Louis (WUSTL), which includes matched metastatic (M) and primary (P) samples, was assessed for relative contribution of SBS2 and SBS13 (**B**). For patients with metastatic sites that demonstrated > 4% relative contribution (upper quartile) of SBS2 and SBS13 (patient nos. 4, 7, 14, 15, 17, 18), paired P and M SBS2 and SBS13 fractions are shown. (**C**) Survival of patients with tumors designated as APOBEC3 high (>4%) versus low (<4%). (**D**) Patients were grouped by long-term (>5 years) and short-term (<3.5 years) survival. The relative contribution of SBS2+13 in metastatic sites relative to matched primary is shown for individual patients as a heatmap. (**E**) Analysis of relative contribution of SBS2 and SBS13 shows increased SBS2+13 in primary and metastatic sites for individual patients is shown. Blue dots are from patients with short-term survival, and purple dots represent patients with long-term survival. Statistical significance was determined by uncorrected Fisher’s test, *P* = 0.02. (**F**) Genomic assessment of multisite biopsies from patients in the DFCI/PENN cohort. Seven patients with >4% SBS2 and SBS13 relative contribution are shown. Relative contribution of SBS2 and SBS13 shown for each biopsy site ranging from precursor lesions (p53 signatures, serous tubal intraepithelial carcinoma [STIC]) to metastatic sites. Three patients had biopsies of only STIC lesions (64, 65) (patient genome 304). Unlabeled circles and arrows between lesions indicate inferred subclonal hierarchy, adapted from Labidi-Galy, et al. (41), and predicted progression of disease.

To determine the prevalence of APOBEC3 activity in HGSOC, we assessed genome sequencing from 3 previously published HGSOC datasets: Pancancer Analysis of Whole Genomes (PCAWG) (40), The Cancer Genome Atlas (TCGA), and Dana Farber Cancer Institute/University of Pennsylvania cohort (DFCI/Penn) (41). For each genome, we assessed the contribution of SBS signatures defined by COSMIC. When averaged across each dataset, we found that the APOBEC3 mutational signatures, SBS2 and SBS13, comprised 4.5%–6.5% of the mutational burden in HGSOC (Figure 1A and Supplemental Figure 1A; supplemental material available online with this article; https://doi.org/10.1172/jci.insight.186409DS1). APOBEC3 mutational signatures did not correlate with specific genetic (i.e., homologous recombination deficiency, cyclin E amplification) or molecular subtypes (42) but were evident in slightly less than half of all HGSOC genomes analyzed (Supplemental Figure 2).

In a previous pan-cancer study, APOBEC3 mutagenesis was determined not to be significantly different between primary tumors and metastatic sites (28). To query whether differences in APOBEC3 activity exist between primary and metastatic sites in HGSOC, we took advantage of a dataset from Washington University in St. Louis (WUSTL; St. Louis, Missouri, USA), which includes paired primary and metastatic whole exome sequencing (WES) from 35 patients with FIGO stage III–IV HGSOC prior to treatment (43). When pooling all primary and metastatic samples, we observed that SBS2 and SBS13 were increased in metastatic sites by 6-fold relative to primary sites (Supplemental Figure 1B). We then looked at the individual patients from the WUSTL cohort with the highest contribution of APOBEC3 signature mutations to tumor mutational burden. Six of 35 patients (5.8%) exhibited greater than 4% contribution of APOBEC3 signature mutations in metastatic tumor genomes (Figure 1B and Supplemental Figure 1C). All 6 patients had an increase in the contribution of APOBEC3 signature mutations in metastatic sites compared with primary tumors (Figure 1B). To delineate which APOBEC3 enzyme was most likely responsible for this mutagenesis, we analyzed the extended sequence context in which cytosines were mutated within cancer genomes. Prior studies have demonstrated that A3A mutagenesis occurs within a YTCA sequence context (Y=T or C), whereas A3B acts more frequently at RTCA sequences (R=A or G) (29). We found that mutated cytosines within SBS2/13 signatures in HGSOC genomes more commonly occurred in a YTCA context, consistent with A3A activity (Supplemental Figure 1D).

Given the observed association between APOBEC3 mutagenesis and tumor metastasis, we investigated how APOBEC3 SBS signatures correlated with patient survival. We divided the WUSTL patients by those with high (>4%) or low (<4%) contribution of APOBEC3 signature mutations in metastatic tumors. We found that patients with high APOBEC3 mutagenesis had an average survival of 816.7 days while patients with low APOBEC3 mutagenesis had an average survival of 1,655.3 days, correlating a high burden of APOBEC3 SBS signatures with decreased overall survival (Figure 1C). We next analyzed patients in the WUSTL cohort by categorizing them as short-term (<3.5 years) or long-term (>5 years) survivors (43). We identified that APOBEC3 SBS signatures were more abundant in tumor genomes from short-term compared with long-term survivors (Figure 1D). Upon examination of primary-metastatic pairs, we found that APOBEC3 SBS signatures were enriched in the metastatic sites of short-term survivors compared with long-term survivors (Figure 1E). These data indicate that APOBEC3 mutagenesis is enriched in metastatic HGSOC and correlated with decreased patient survival.

As APOBEC3-mediated mutagenesis increased from primary to metastatic tumors, we sought to determine the kinetics of APOBEC3 activity throughout HGSOC evolution. Within the DFCI/Penn cohort, we assessed multisite biopsies from patients ranging from normal fallopian tube tissue, *TP53-*mutant single-cell epithelial layer (p53 signature), serous tubal intraepithelial carcinoma (STIC), and primary and metastatic HGSOC lesions (41). In addition, 3 patients with only STIC lesions were analyzed for mutational signatures relative to normal fallopian tube tissue. Of 9 patients in the cohort, 7 had measurable APOBEC3 mutational signatures in at least 1 tumor site (Figure 1F). Interestingly, all 7 patients in whom APOBEC3 mutational signatures were evident had measurable APOBEC3 mutational signatures early in tumor development at the STIC lesion stage, with contributions ranging from 7% to 21%. Four of the DFCI/Penn patients had sequenced biopsies of samples beyond the STIC lesion stage, and 3 of 4 exhibited persistence or increase of APOBEC3 mutagenesis as tumors evolved (9.5%–29% contribution; Figure 1F), consistent with the increased APOBEC3 activity observed in the WUSTL cohort metastatic lesions. These data indicate that APOBEC3 mutagenesis may arise early in HGSOC development but can accelerate or accumulate throughout tumor evolution. In a minority of tumors, APOBEC3-mediated mutagenesis appears not to expand in metastatic lesions. Together, findings from primary tumor genomes demonstrate relatively frequent A3A mutagenesis in HGSOC that is enriched in metastatic sites and associated with poor survival, suggesting that A3A activity enables tumor progression.

### Modeling episodic A3A expression in HGSOC cells.

**Figure 2:**
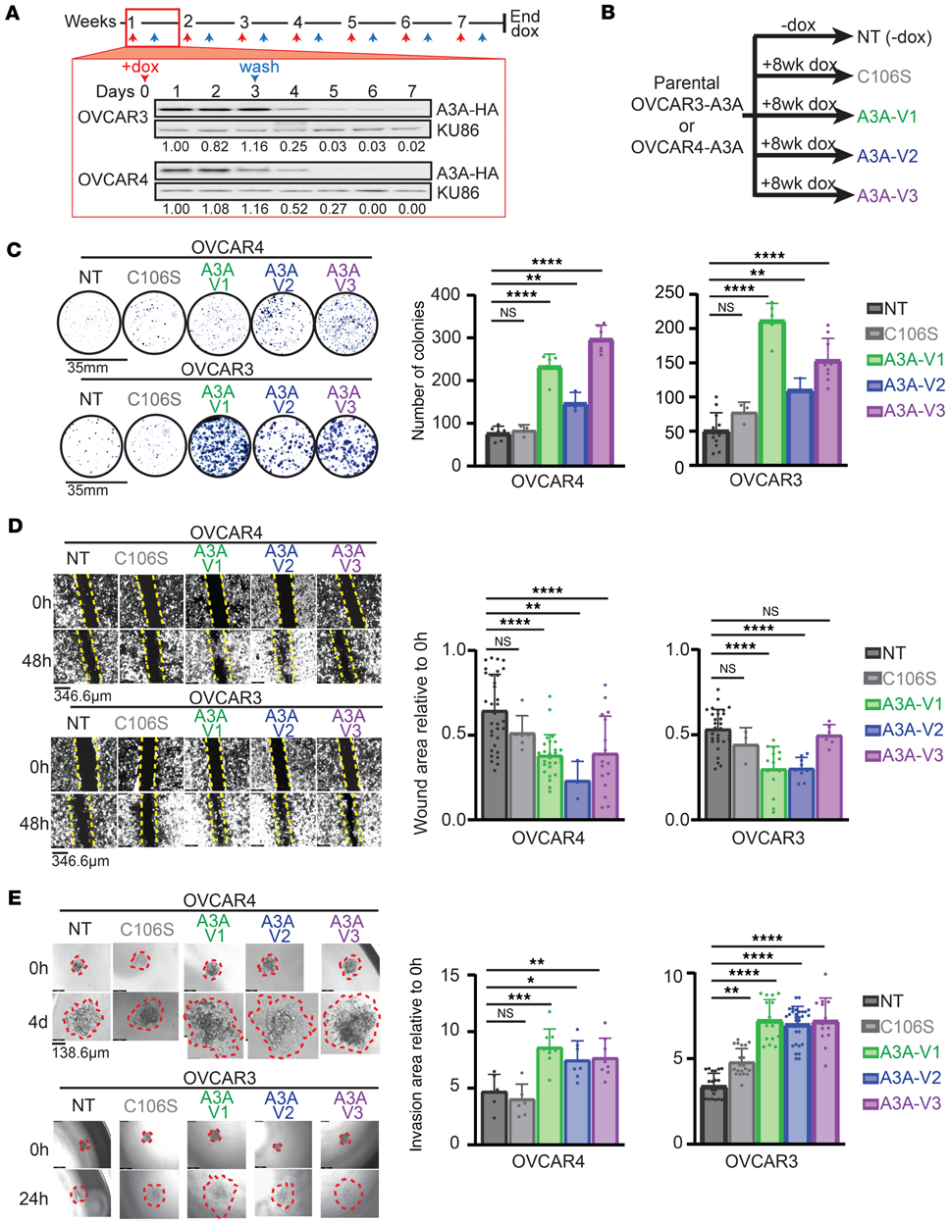
Episodic A3A expression promotes HGSOC cell survival, migration, and invasion. (**A**) OVCAR3 and OVCAR4 cells engineered to stably express a dox-inducible *A3A* transgene were treated with dox on day 0 and washed out on day 3. Treatment schema was repeated for 8 weeks. Immunoblot of HA-tagged A3A from cell lysates harvested on sequential days throughout 1 week following dox treatment. Ku86 and H3 are loading controls. Bands are quantified relative to loading control and normalized to day 1 lane. (**B**) Three biological replicates of A3A cell lines (A3A V1–V3) and a cell line induced to express a catalytic mutant of A3A (C106S) were independently derived from the parental cell lines (NT). NT cells were cultured in parallel for 8 weeks. (**C**) Cell survival under stress was assessed using colony formation assays. Cells were seeded at ultra-low cell densities and resulting colonies were then stained, imaged, and quantified. (**D**) Wound healing assays were performed to assess the migratory phenotype of OVCAR3 and OVCAR4 NT, A3A V1–V3, and C106S cells. The wound was imaged using a 4× objective at 0 hours and 48 hours. Wound area at 48 hours relative to 0 hours is shown in bar graph. (**E**) Spheroids of each cell line were generated and placed onto a Matrigel-containing pseudo–basement membrane. Spheroids were imaged with a 10× objective at 0 hours, 24 hours, and 4 days. Area of the spheroid at 24 hours or 4 days relative to 0 hours is shown. Invasion area is outlined in red. For data in **C**–**E**, representative images are shown and quantification is depicted in bar graphs below. Significance was determined by 1-way ANOVA with Dunnett’s correction for multiple comparison, ****P ≤ 0.0001, ***P ≤ 0.001, **P ≤ 0.01, *P ≤ 0.05. Data are shown as mean ± SD for *n* ≥ 3 replicate experiments. Representative images are shown.

To experimentally determine how APOBEC3 mutagenesis affects ovarian cancer progression, we developed cellular models of APOBEC3 expression in HGSOC. We utilized 2 HGSOC cell lines to model episodic A3A activity. *A3A* is an IFN-stimulated gene (44), and we selected OVCAR3 and OVCAR4, which do not express A3A even when stimulated with IFN (Supplemental Figure 3). Importantly, both cell lines have *TP53*-inactivating mutations, consistent with the designation of HGSOC. We introduced a doxycycline-inducible (dox-inducible) *A3A* transgene by lentiviral integration into both cell lines (OVCAR3-A3A and OVCAR4-A3A), enabling controllable A3A expression and activity (Figure 2A and Supplemental Figure 4). A3A mutagenesis has been shown to occur in intermittent bursts over time (45, 46). To mimic intermittent A3A expression in cancer, we treated cells once weekly with low-dose (0.5 mg/mL) dox followed by dox washout 72 hours later (Figure 2A). This approach resulted in A3A expression after 1 day of treatment that gradually led to undetectable levels by day 5 of treatment (Figure 2A). We repeated this treatment course every 7 days for 8 weeks, after which we initiated experimental investigations. This treatment schema provided a system to address how the consequences of historic, intermittent A3A expression, rather than ongoing A3A mutagenesis, affects tumor cell phenotype. To account for the previously established stochastic nature of A3A activity (7), we generated 3 independent replicates of each cell line (A3A V1, A3A V2, A3A V3) (Figure 2B). All A3A replicates were derived from nontreated (NT) control cells, which underwent viral integration of the transgene but were never exposed to dox or A3A expression (Figure 2B). A prior report suggested that A3A expression, but not activity, influenced survival and metastatic spread of pancreatic cancer (47); thus, we generated OVCAR3 and OVCAR4 cells with catalytically inactive *A3A* transgenes (A3A-C016S) and subjected them to the same treatment schema as in Figure 2A to enable assessment of deaminase activity.

### Episodic A3A deaminase activity promotes HGSOC cell survival, migration, and invasion.

Since APOBEC3 mutagenesis in patient tumors was correlated with decreased survival (Figure 1, C–E), we hypothesized that A3A would promote phenotypic changes consistent with increased metastatic potential. We examined 3 key steps in metastasis — cell survival, migration, and invasion — in A3A-exposed cells. We assessed the ability of HGSOC cells to survive under stress by seeding at ultra-low densities. We found that OVCAR3-A3A and OVCAR4-A3A cells formed significantly more colonies than control (NT) cells (Figure 2C). Increased colony formation was not due to an altered rate of proliferation, since A3A and NT HGSOC cells grew at similar rates (Supplemental Figure 5). In addition, using wound-healing assays, we found that OVCAR3-A3A and OVCAR4-A3A cells migrated more rapidly into a wound than NT controls, indicating enhanced migratory phenotypes (Figure 2D). We analyzed whether A3B expression elicited the same effect and found no significant differences in wound closure after intermittent A3B expression in OVCAR3 cells relative to NT controls (Supplemental Figure 6).

Finally, we assessed how episodic A3A expression affects the invasive potential of HGSOC cells. We generated spheroids of each cell line and placed them in a pseudo–basement membrane (0.5 mg/mL Matrigel). By serial imaging, we found that A3A-exposed OVCAR3 and OVCAR4 cells demonstrated a greater area of invasion into a pseudo–basement membrane than NT controls (Figure 2E). Notably, we found that all OVCAR3-A3A and OVCAR4-A3A cell lines (V1–V3) exhibited similarly altered phenotypes. Importantly, cells exposed to intermittent A3A-C106S expression did not exhibit increased survival, migration, or invasion (Figure 2, C–E), indicating that catalytic activity of A3A is required for this phenotype. Together these data show that episodic A3A activity promotes protumor, metastatic phenotypes in culture indicated by increased survival, migration, and invasion.

### A3A accelerates distant HGSOC metastasis in vivo.

**Figure 3:**
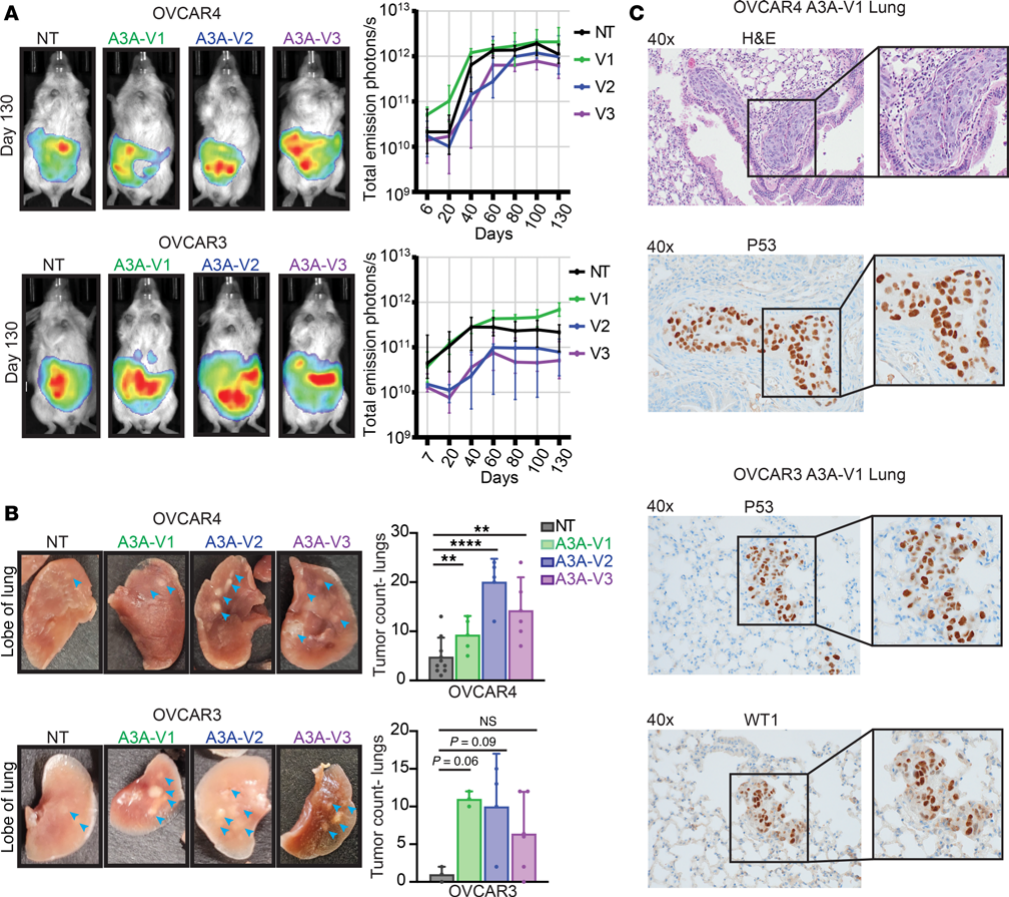
A3A promotes distant HGSOC metastasis in vivo. (**A**) Representative images of total emission in photons/sec of BLI from day 130 after injection. BLI over time plotted as total emission in photons/sec for each time point; data are shown as median ± SD. (**B**) Lungs were harvested from the mice at death or 165 days after injection, and tumor burden was determined by macroscopic count of tumor nodules. Representative images are shown; blue arrows indicate macroscopic tumor sites. Significance determined by 1-way ANOVA with Dunnett’s multiple comparison correction. Data are shown as mean ± SD for duplicate experiments (*n* = 3–5 mice for each experiment). ****P ≤ 0.0001, **P ≤ 0.01. (**C**) Lungs were sectioned and analyzed by H&E staining, and IHC staining for TP53 and Wilms’ tumor-1 (WT1). Representative images are shown at 40×.

We reasoned that the phenotypic changes observed in HGSOC cells in culture would affect tumor metastases in vivo. As ovarian cancer progresses in patient tumors, clusters of cancer cells detach from the primary tumor into the peritoneal cavity and seed both local and distant sites (48). Thus, we designed in vivo experiments to mimic ovarian cancer progression in patients by engrafting OVCAR3 or OVCAR4 cell clusters suspended in 0.5 mg/mL Matrigel into the peritoneal cavity of immunodeficient mice. This protocol enabled the formation of tumor spheroids immediately after injection, replicating the behavior of tumor clusters that have migrated from the primary tumor site in patients (48, 49). After delivery of the OVCAR3 or OVCAR4 cells, we monitored tumor burden via bioluminescent imaging (BLI) and found stable engraftment of all cell lines (NT and A3A V1–V3) within 2–3 weeks of injection (Figure 3A). Following stable engraftment of tumor in the peritoneal cavity, we monitored tumor burden through serial BLI for 165 days and found no differences in overall tumor burden between A3A-exposed and NT control xenografts (Figure 3A). Both BLI and postmortem measurement of tumor weights demonstrated a high burden of disease within the peritoneal cavity, consistent with multifocal seeding from i.p. tumor injection (Figure 3A and Supplemental Figure 7).

We next examined extraperitoneal organs to evaluate distant metastasis. Through macroscopic assessment of tumor nodules in the lungs, we found that mice engrafted with A3A-exposed OVCAR3 and OVCAR4 cells had significantly more metastatic seeding in the lungs than NT controls (Figure 3B). Histopathologic examination and IHC staining for WT1 and p53 confirmed the presence of ovarian tumor within the lungs (50) (Figure 3C). These data demonstrate that episodic A3A activity in HGSOC cells enhances tumor metastasis to distant sites.

### Episodic A3A activity in HGSOC cells leads to stochastic mutagenesis.

**Figure 4:**
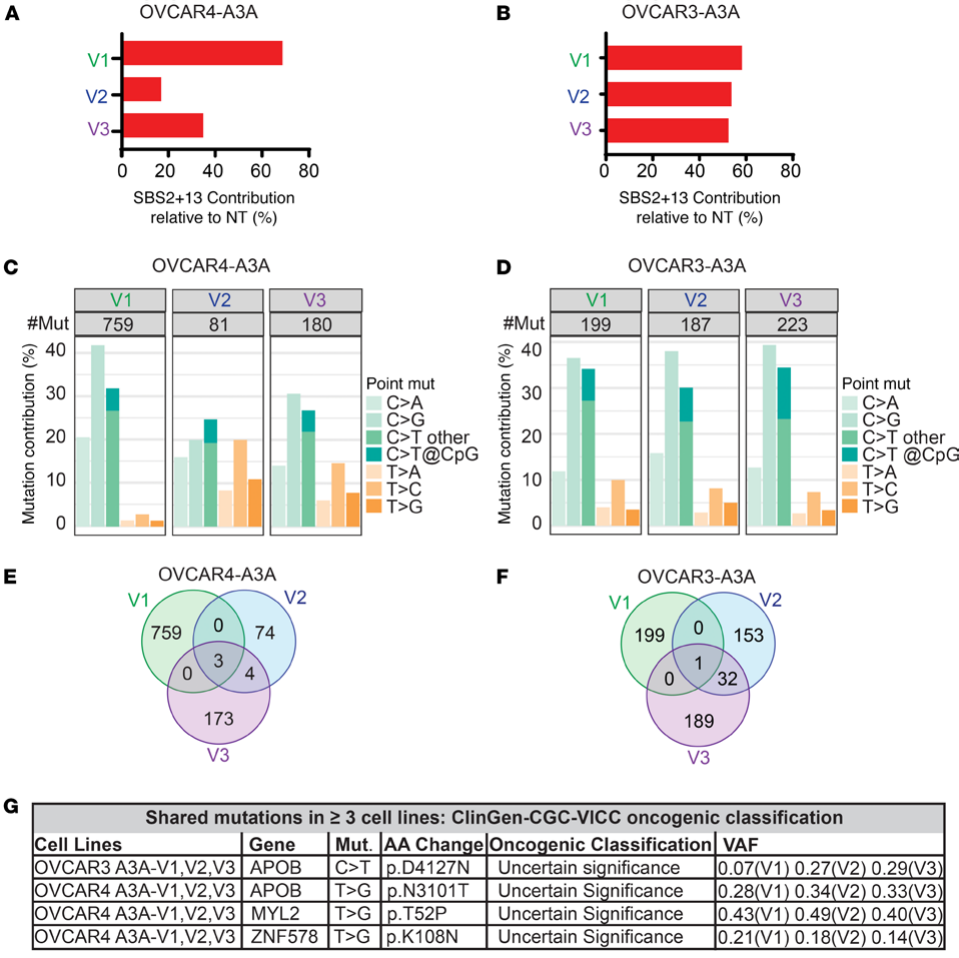
Episodic A3A in HGSOC causes stochastic mutagenesis. Whole exome sequencing of OVCAR4 and OVCAR3 A3A V1–V3 and NT cell lines was performed. Respective NT cell lines were used as a reference genome to determine de novo mutations in A3A-exposed cells. (**A** and **B**) Contribution of SBS2 and SBS13 to total de novo mutations. (**C** and **D**) The total number of de novo base substitution mutations and relative contribution of transition and transversion mutations. Total number of acquired mutations is shown for each version. (**E** and **F**) Venn diagram of mutations acquired in A3A-exposed OVCAR4 and OVCAR3 cell lines versions 1–3. (**G**) ClinGen-CGC-VICC was used to identify the oncogenic classification of mutations acquired in ≥ three A3A-exposed cell lines. The cell line, mutated gene, base substitution, amino acid change, oncogenic classification, and variant allele frequency (VAF) are shown.

Given the distinct prometastatic phenotype of A3A-exposed HGSOC cells in culture and in vivo, we sought to identify mechanisms underlying A3A-mediated tumor behavior. First, to determine how A3A activity affects the genomes of OVCAR3 and OVCAR4 cells, we performed deep WES (~200× coverage) on NT and A3A V1–V3. Using NT cells as reference genomes, we assessed how the mutational burden was altered by episodic A3A expression. We found that SBS2 and SBS13 signatures contributed to ~20%–70% of the potentially novel mutational burden after episodic A3A exposure (Figure 4, A and B). While the fraction of SBS2 and SBS13 contributing to the overall mutational burden in these cells is higher than we report for patient tumors (Figure 1 and Supplemental Figure 1), the total number of mutations caused by A3A (range, 81–759) are analogous to those reported in patient tumor sequencing (average of 151 mutations/tumor attributable to SBS2 and SBS13 in ovarian cancers from COSMIC) (Figure 4, C and D) (17).

Next, we assessed the potentially novel mutations that were acquired in A3A-exposed cells. We found variation in the number of mutations across versions (Figure 4, C and D). The mutations acquired were largely unique to each version (Figure 4, E and F, and Supplemental Table 1), consistent with prior findings that A3A acts stochastically on the genome (7, 30, 45). In OVCAR4-A3A V1–V3, only 7 mutations were shared between at least 2 versions and none were classified as likely to be oncogenic by ClinGen-CGC-VICC (51) (Figure 4, E and G, and Supplemental Table 1). In OVCAR3-A3A V1–V3, we found 33 mutations shared among at least 2 versions, none of which were classified as oncogenic (Figure 4, F and G, and Supplemental Table 1). These data suggest that A3A-induced mutations in protein-coding regions did not explain the shared prometastatic phenotypes observed in A3A-exposed cells.

### Episodic A3A expression alters the EMT trajectory of HGSOC.

**Figure 5:**
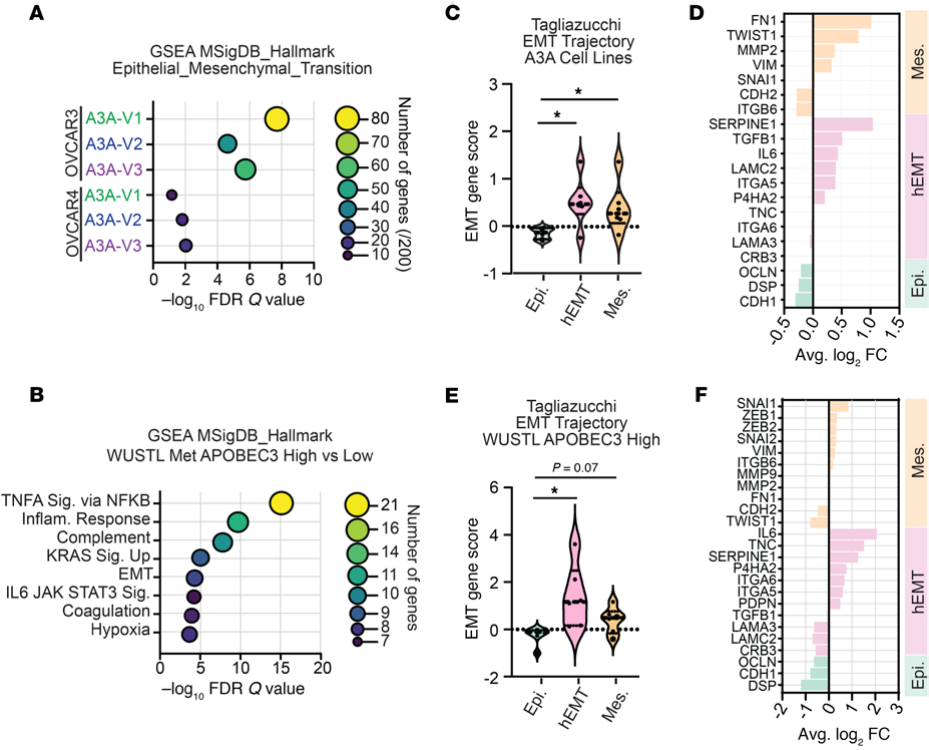
Episodic A3A alters epithelial-mesenchymal transition in HGSOC. (**A**) RNA-Seq from OVCAR4 and OVCAR3 A3A V1–V3 and NT parental cell lines was performed. Gene set enrichment analysis (GSEA) of the significant differentially expressed genes (DEGs) between A3A-exposed and NT cells demonstrated that the most significantly enriched MSigDB Hallmark gene set for each version was Hallmark EMT. Significance determined by FDR *q* value. Color and size of bubbles in plot indicates number of DEGs identified in the Hallmark EMT gene set (out of 200 genes total in gene set) for each A3A-exposed cell line. (**B**) RNA-Seq from the WUSTL patient cohort was analyzed to determine DEGs between metastatic samples with low (<4%) or high (>4%) contribution of APOBEC3 mutational signatures. Significantly different gene sets were determined by FDR *q* value. Color and size of bubbles in plot indicates number of DEGs identified in each significant Hallmark gene set. (**C**) DEGs were assessed using the Tagliazucchi EMT trajectory score (38) to define epithelial (Epi.), hybrid (hEMT), and mesenchymal (Mes.) macrostates in each cell line. A3A V1–V3 for both cell lines are grouped together. Violin plots show the score for each EMT category. Values less than 0 indicate underrepresented gene groups. Significance determined by Brown-Forsythe and Welch 1-way ANOVA with Dunnett’s T3 correction for multiple comparison. *P ≤ 0.05. (**D**) Bar graph showing the DEGs in OVCAR-A3A cells found in the Tagliazucchi EMT trajectory score. (**E**) The Tagliazucchi EMT trajectory was applied to DEGs from metastatic tumors from WUSTL cohort patients with APOBEC3 high versus low mutational burdens. Violin plots show the score for each EMT category. Values less than 0 indicate underrepresented gene groups. Significance determined by 2-tailed *t*-test. **P ≤ 0.01. (**F**) Bar graph of WUSTL cohort DEGs from the Tagliazucchi EMT trajectory score.

Given the lack of shared aberrations in the protein-coding genome to explain the A3A-mediated metastatic phenotype, we sought to assess transcriptional changes that may provide a mechanism for the consistent phenotypic changes observed across A3A-exposed HGSOC cells. From RNA-Seq, we identified differentially expressed genes (DEGs) in A3A-exposed cells relative to NT controls (Supplemental Figure 8, A and B). We additionally assessed DEGs from metastatic patient tumors in the WUSTL dataset by comparing those with high versus low burdens of APOBEC3 mutagenesis as defined in Figure 1C. Through gene set enrichment analysis (GSEA), we determined that DEGs in all A3A cell versions were significantly enriched for genes associated with EMT (Figure 5A, Supplemental Figure 9, and Supplemental Tables 2 and 3). We found a similar enrichment of EMT genes in metastatic tumors from patients in the WUSTL dataset that had APOBEC3-high relative to -low mutational burden (Figure 5B and Supplemental Tables 2 and 3).

EMT is a spectrum of transcriptomic and phenotypic states, the consequences of which vary with regard to progression of HGSOC (38, 52–54). We utilized a previously defined EMT gene score (38, 53) to identify how transcriptional changes resulted in epithelial, mesenchymal, or hEMT macro-states within OVCAR3 and OVCAR4 cells exposed to episodic A3A. This analysis demonstrated that exposure to episodic A3A shifted OVCAR3 and OVCAR4 cells away from epithelial states toward hEMT and mesenchymal cell states (Figure 5, C and D). In addition to the designated EMT gene set, we identified upregulation of EMT-associated gene sets in OVCAR3 and OVCAR4 cells after episodic A3A expression, including TNF-α/NF-kB signaling, IL-6/JAK/STAT3 signaling, and inflammatory responses (Supplemental Figure 9). Using RNA-Seq from patients with HGSOC in the WUSTL cohort, we applied the EMT gene score to metastatic tumors with low or high levels of APOBEC3 mutagenesis (Figure 5E). We found that high levels of APOBEC3 mutagenesis correlated with an increase in both hEMT and mesenchymal genes in metastatic HGSOC sites (Figure 5, E and F). These data show a correlation between APOBEC3 activity and altered EMT in both metastatic patient tumors and experimental models of HGSOC.

**Figure 6:**
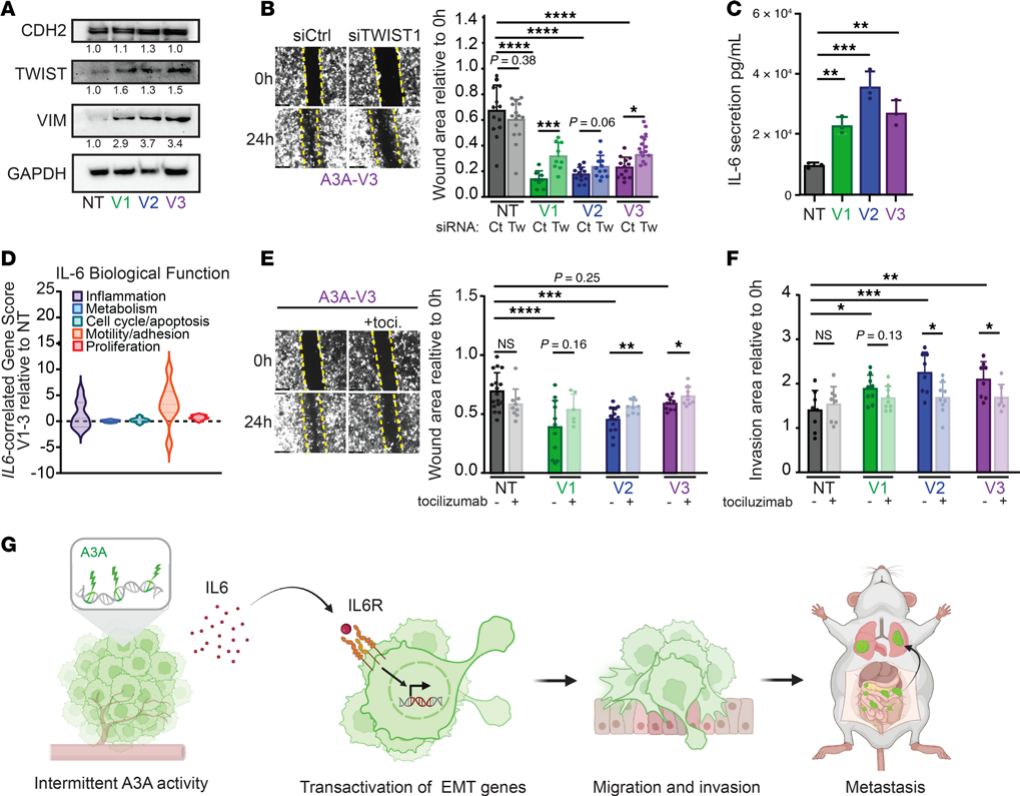
Inhibition of EMT signaling limits A3A-induced migratory phenotype. (**A**) Immunoblot of mesenchymal markers CDH2, TWIST1, and Vimentin in OVCAR3 A3A V1–V3 and NT cells. GAPDH is a loading control. Bands are quantified relative to loading control and normalized to NT lane. (**B**) Wound healing assay of OVCAR3-A3A NT and V1–V3 cells depleted of TWIST1 (Tw) by siRNA or transfected with nontargeting siRNA control (Ct). Images were acquired with 4× objective; representative images from V3 are shown. Wound area was calculated and is plotted as 24-hour area relative to 0 hours. Yellow lines indicate region of original wound (0 hours). (**C**) ELISA quantification of IL-6 in the media of OVCAR3-A3A V1–V3 and NT cell lines after 3 days in culture. (**D**) *IL6*-correlated gene score (67) assessed by RNA-Seq of OVCAR3-A3A NT and V1–V3 cell lines. (**E**) Wound healing assay of OVCAR3-A3A V3 cell lines after 24 hours of treatment with tocilizumab. Wound area was calculated and is plotted as 24-hour area relative to 0 hours. Wound size at 0 hours is outlined in yellow. (**F**) Spheroids of each cell line were generated and placed onto a Matrigel-containing pseudo–basement membrane. Spheroids were imaged with a 10× objective at 0 hours and 48 hours. Area of the spheroid relative to 0 hours is shown. For **B**–**F**, comparison between multiple groups was determined by ordinary 1-way ANOVA with Dunnett’s correction for multiple comparison. For comparison between 2 groups, significance was determined by unpaired *t* test. *P ≤.0.05, **P ≤ 0.01, ***P ≤ 0.001, ****P ≤ 0.0001. Data are shown as mean ± SD for *n* ≥ 3 biological replicates. (**G**) Model of A3A-mediated metastatic progression. Intermittent A3A activity results in increased IL-6 secretion and transactivation of *IL6*-associated EMT gene programs, activation of migration and invasion, and distant metastatic spread.

We next analyzed EMT protein expression and found upregulation of mesenchymal markers (CDH2, VIM, TWIST1; Figure 6A and Supplemental Figure 10A). We hypothesized that gene and protein expression changes indicative of hEMT/mesenchymal phenotypes were responsible for the migratory phenotypes observed in A3A-exposed HGSOC cells and xenografts (Figure 2 and Figure 3). We performed siRNA knockdown of the mesenchymal marker TWIST1. TWIST1 is a highly conserved transcription factor that plays a prominent role in EMT (55–63) and was upregulated in HGSOC cells exposed to episodic A3A (Figure 5D, Figure 6A, and Supplemental Figure 10A). We depleted TWIST1 in OVCAR cells and found that migration into a wound was significantly inhibited in A3A-exposed cells, whereas NT controls were unaffected (Figure 6B and Supplemental Figure 10B).

The cytokine IL-6 is secreted by cancer cells undergoing EMT and, in turn, can activate EMT plasticity (64–66). We noted enrichment of IL-6 signaling in RNA-Seq from metastatic patient tumors (Figure 5, B and F) and OVCAR cell lines (Figure 5D and Supplemental Figure 9) with high levels of APOBEC3 mutational signatures. In addition, we found increased IL-6 secretion by OVCAR3 and OVCAR4 cells after episodic A3A expression relative to NT controls (Figure 6C and Supplemental Figure 10C), indicating a potential mediator of EMT. IL-6 has been associated with HGSOC progression (67, 68). A prior study developed an *IL6*-correlated gene score by assessing genes coexpressed with IL-6 in HGSOC (67). *IL6*-correlated genes were involved in discreet cellular processes including proliferation, inflammation, and motility/adhesion. We applied the *IL6*-correlated gene score to DEGs in OVCAR-A3A cells and found that coexpressed genes fell largely into the motility/adhesion category, consistent with the migratory phenotype we observed in OVCAR cells after intermittent A3A expression (Figure 6D and Supplemental Figure 10D). To assess the necessity of IL-6 signaling in EMT alterations noted in HGSOC cells with A3A expression, we used tocilizumab to block the IL-6 receptor. In OVCAR3 cells, we noted that IL-6 blockade reversed the migratory phenotype noted after A3A expression (Figure 6, E and F). Taken together, these data suggest a model in which intermittent A3A activity in HGSOC cells results in upregulation of *IL6*, and signaling through the IL-6 receptor on HGSOC cells results in transactivation of EMT genes, leading to mesenchymal transition and enhanced metastatic potential (Figure 6G).

## Discussion

Despite advances in diagnostic and therapeutic approaches, HGSOC remains the most lethal gynecologic malignancy, largely due to its propensity for metastasis and chemoresistance (2–5). Utilizing unique precursor, primary, and metastatic cancer samples from individual patients, we found a substantial contribution of APOBEC3 mutagenesis in HGSOC genomes, particularly in metastatic sites. We additionally established a correlation between high APOBEC3-induced mutagenesis and decreased patient survival. Previous studies correlating elevated expression of A3B in HGSOC and patient survival reported conflicting results (9, 69). However, recent reports indicate that A3A is the more frequent contributor to APOBEC3-mediated mutations in human tumors (8, 30). We demonstrated that A3A expression in HGSOC cells promoted metastatic phenotypes and expression of EMT pathways in a deaminase-dependent manner. Notably, our murine studies revealed that A3A expression enhanced distant metastatic seeding in the lungs. Thus, we defined both in patient tumors and experimental models how A3A is associated with HGSOC progression.

The genomic landscape of HGSOC is defined by a number of recurrently mutated genes, most notably *TP53* and *BRCA1/2* (43, 70, 71). However, drivers of disease progression and chemoresistance remain enigmatic. Comparison of metastatic to primary HGSOC has revealed an increase in tumor mutational burden and overall genomic instability as tumors progress (72). Here we define cytidine deamination by APOBEC3 enzymes as a mediator of mutagenesis in HGSOC, particularly in the progression from primary to metastatic tumors. We found that mutations acquired in HGSOC cells exposed to A3A recapitulated previously reported mutations in patients with HGSOC, notably in BRCA2 *BRCA2* (72), *LEPR* (73), *NSD2* (74), *ITGB2* (75), and *TTN* (76) (Supplemental Table 1). Interestingly, we detected a previously identified hotspot for A3A mutagenesis in the helical domain of PIK3CA (c.1624G > A; c.1633G > A) (12, 77) (Supplemental Table 1), which is typically associated with clear cell ovarian carcinoma. Several pathogenic gene mutations attributable to A3A in our study are less frequently reported in patients with HGSOC (*TNRC6A*, *BMPR1B*, *JARID2*, *CUX1*, *PRKCG*, *LAMP2*; Supplemental Table 1). Our findings of variable mutations even in isogenic models are consistent with the stochastic nature of A3A activity (7, 30) and indicate that A3A may contribute to the well-established inter- and intratumor heterogeneity of HGSOC (78–81).

Despite the diversity in the total number and location of mutations mediated by A3A activity across cell lines, we found similar alteration in phenotypes of HGSOC cells. HGSOC cells exposed to A3A and patient tumors with high levels of APOBEC3-mediated mutagenesis exhibited consistent alterations in EMT gene expression. EMT pathways are essential in nonmalignant cells during development and wound healing (37). Interestingly, a recent analysis of differentiating keratinocytes demonstrated that upregulation of A3A occurred simultaneously with genes involved in the wound-healing response (46). While previous studies have identified tumor samples in which both APOBEC3 mutational signatures and EMT transcriptional profiles exist concurrently (38, 82, 83), an experimental link between A3A and EMT has not been previously reported. EMT plasticity, in which tumor cells move fluidly between epithelial and mesenchymal phenotypes, is an established mode of ovarian tumor progression and metastasis (38, 39, 48, 52, 84, 85). Importantly, HGSOC cells exhibit transcriptional profiles consistent with a combination of epithelial and mesenchymal phenotypes (hEMT) that correlates with more aggressive disease states (38, 54). Here we found that APOBEC3 activity led to hEMT/mesenchymal transcriptional profiles in HGSOC cells and metastatic patient tumors. Among other hEMT markers, we found upregulation and secretion of IL-6, which itself is an inducer of migratory phenotypes in HGSOC (66, 86). A3A expression has been correlated with *IL6* expression in cancer cell lines and primary macrophages (87, 88). Therefore, a potential model to explain the correlation between A3A and EMT is through A3A-mediated upregulation of *IL6*, which promotes induction of EMT (Figure 6G). Our study lays a foundation to study the transcriptional circuits underlying A3A-mediated EMT and how A3A affects EMT in other tumor types.

The molecular mechanism by which A3A activity elicits EMT transcriptional programs was not explained by recurrent genomic mutations found in HGSOC cell lines in this study. However, it is possible that transcriptional alterations are generated by deaminase-induced changes to chromatin function. In contrast to a prior study that suggested that A3A could promote metastatic spread of pancreatic cancer through deaminase-independent functions (47), we show that the deaminase activity of A3A is required to promote metastatic phenotypes in ovarian cancer. A3A can act on methylated cytidines (5-methylcytosine [5mC]), resulting in the direct conversion of 5mC to thymidine and loss of the DNA methylation mark (7, 89–91). Global alterations in DNA-methylation patterns, which canonically repress transcription, may affect chromatin accessibility and gene expression. Interestingly, *IL6* expression was previously shown to be upregulated in HGSOC by epigenetic alterations leading to increased chromatin accessibility (86). A3A activity may similarly affect *IL6* expression through epigenetic mechanisms.

Alternatively, A3A binding to promoters may affect transcriptional control. A recent study found that A3A binds the TTTC motif within IFN-stimulated response elements (IRSE), which limits IFN-stimulated gene expression (92). A similar mechanism of A3A binding to regulatory elements may influence the expression of EMT genes. It is also possible that A3A-induced DNA breaks or methylation changes may disrupt or create transcription factor binding sites. A study of the related enzyme, A3B, in breast cancer found that deaminase activity at estrogen receptor (ER) binding sites resulted in DNA breaks that, upon repair, remodeled chromatin locally to promote ER-dependent gene expression (93). A3A may induce similar chromatin alterations through damage to the genome that ultimately affects EMT gene expression. An important outstanding question is whether transcriptional alterations driven by A3A are durable even in the absence of ongoing A3A expression or activity, and this may illuminate the potential mechanisms by which A3A promotes gene expression changes. Further research into the dynamics of chromatin architecture and epigenetic modifications influenced by A3A activity is warranted.

In conclusion, our study highlights the effect of A3A activity on HGSOC progression through promotion of metastasis via EMT. By integrating genomic analyses of primary and metastatic HGSOC tumors with experimental models, we provide mechanistic insights into the role of A3A in driving HGSOC metastasis. Our findings provide a foundation for investigating A3A as a predictive or prognostic biomarker and for identifying novel therapeutic approaches aimed at mitigating the consequences of aberrant A3A activity.

## Methods

Supplemental Methods are available online with this article.

### Sex as a biological variable

In this study, we exclusively studied tumor genomes from women and used female mice as experimental subjects because ovarian cancer, the focus of our research, is a disease unique to females due to its origin in the female reproductive system.

### Whole-exome sequencing

Genomic DNA was extracted using the PureLink Genomic DNA Mini Kit (Invitrogen). Library preparation and whole-exome sequencing (WES) was performed at Washington University School of Medicine Genome Technology Access Center (GTAC). gDNA (100–250 ng) was fragmented using a Covaris LE220 to achieve an approximate size of 200–375 bp. Libraries were constructed using the KAPA Hyper Prep Kit (KAPA Biosystems, 7962363001) on a Perkin Elmer SciCloneG3 NGS (96-well configuration) automated workstation. Individual libraries were pooled for capture at an equimolar ratio yielding up to 5 μg per pool and hybridized with the xGen Exome Research Panel v2.0 (IDT Technologies) capture reagents. The libraries contain custom Illumina adapters with 10 base dual unique indexes. Pooled libraries were sequenced to generate paired end reads of 151 bases using an Illumina NovaSeq X plus instrument. Base calling and demultiplexing (to create sample-specific FASTQ files) were performed with the BCL Convert utility. This processing was performed with onboard software or off-instrument using a stand-alone DRAGEN processor using the same algorithm.

#### Alignment of WES reads and mutation calling.

All samples were analyzed using a DRAGEN BioIT processor running software version 4.2.4 (Illumina) in tumor-normal mode. For all OVCAR4 and OVCAR3 WES, sequencing reads were aligned to GRCh38 reference genome and outputted in the CRAM format. To determine potentially novel mutations acquired after A3A expression, OVCAR4-A3A V1–V3 and OVCAR3 V1–V3 were compared with isogenic NT controls, and any shared mutations were filtered out. The oncogenicity of shared variants between at least 2 samples was accessed according to the ClinGene-CGC-VICC classification guidelines, via Franklin by Genoox (https://franklin.genoox.com) (51). The variants in matched primary tumors and metastases from the WUSTL cohort were analyzed using the pipeline discussed above. To determine acquired mutations that were unique to disease progression, metastases were compared with their matched primary tumors. The DFCI/Penn cohort was assessed for variants as previously described (41).

### Mutational signature extraction from human cancer sequencing

Human cancer data collected from the PCAWG and TCGA databases were obtained from syn11726616, the ICGC Data Portal (release 28), and via TCGAbiolinks, respectively. Information about the TCGA subgroups were obtained from the cBioPortal. De novo APOBEC3 and background spectra were extracted using NMF in the R package MutationalPatterns (v3.3.1) (94). Mutational signatures were deconvoluted to the COSMIC v3.2 reference signature set. Differences in mutational burden and APOBEC enrichment between samples was investigated and visualized using the R packages ggplot2 and tidyverse. Tumors with high APOBEC expression were defined as those in the upper quartile of the WUSTL cohort, which led to a cut off of > 4% SBS2 + SBS13 relative contribution.

### Cell lines and culture

OVCAR4 and OVCAR3 cell lines were provided in house. All cell lines were cultured in DMEM (Thermo Fisher Scientific) + GlutaMAX supplemented with 10% tetracycline-free FBS (MilliporeSigma) and 1% penicillin/streptomycin (Thermo Fisher Scientific). Cells were cultured at 37°C with 5% CO_2._ The creation of OVCAR4-A3A and OVCAR3-A3A cell lines was achieved through lentiviral transduction with the pSLIK-A3A lentivector with neomycin resistance (6). Similar vectors, pSLIK-A3B and pSLIK–A3A-C106S, were used to generate OVCAR-A3B and OVCAR-C106S cells, respectively. Prior to murine experiments, all cell lines were transduced with an EF1α^CBR-GFP^ lentivirus to express Click Beetle Red (CBR) luciferase and GFP, generously provided by the lab of John DiPersio (Washington University School of Medicine in St. Louis). After transduction, cells were sorted via flow cytometry to ensure that the cell population was 100% CBR-GFP positive. Cells were tested for mycoplasma at least every 6 months.

#### Episodic A3A induction.

To achieve episodic A3A expression, 0.5 μg/mL dox (MilliporeSigma) was added to the culture media once per week, with a media change on day 3. The dox treatment regimen was repeated for 7 weeks. Three iterations of each cell line were generated by independent seeding prior to the first dox treatment and parallel culture throughout subsequent treatments (V1–V3).

#### siRNA transfection.

Pooled siRNA oligonucleotides (25 pmol) targeting *TWIST1* (Horizon SMARTpool) were transfected into OVCAR cells using the RNAiMAX transfection reagent (Invitrogen) according to the manufacturer’s protocol. Gene depletion was confirmed by quantitative PCR (qPCR).

### qPCR analysis

RNA was harvested from cell pellets using the Monarch Total RNA Miniprep Kit (New England Biolabs). cDNA was produced using the Invitrogen RNA-to-cDNA kit. qPCR was performed using PowerSyber Green PCR Master Mix (Applied Biosystems) on a QuantStudio 6 Pro (Applied Biosystems) and analyzed by Design and Analysis Software (Thermo Fisher Scientific).

### Immunoblotting

Cell lysates were prepared by boiling in 1X LDS (Invitrogen) for 15 minutes. Once cooled, 20% by volume β-mercaptoethanol was added to the lysates. Samples were run on 10% bis-acrylamide gels in MOPS buffer (Invitrogen) and transferred to nitrocellulose membranes (Cytiva Amersham) using a Bio-Rad turbo blot transfer machine. Blots were blocked in 5% milk and probed with primary antibodies (HA: BioLegend Clone HA.11 [catalog 901514], H3-Abcam [catalog 1791]; CDH2: Sigma-Aldrich [catalog 5600069]; TWIST1: Santa Cruz Biotechnology Inc. [catalog 81417]; Vimentin: Cell Signaling Technology [catalog V6389]; GAPDH: GeneTex [catalog 627408]) overnight. Secondary antibodies for immunoblotting were obtained from Jackson ImmunoResearch (goat anti–rabbit IgG, 111-035-045; goat anti–mouse IgG, 115-035-003). Immunoblots were developed using ECL (Invitrogen) and analyzed on a Bio-Rad ChemiDoc MP.

### Proliferation and colony formation assays

For proliferation assays, cells were seeded in a 24-well plate and harvested and counted in triplicates on days 0, 2, 4, 6, 8, and 10 using the Countess II (Invitrogen). For colony formation assays, 2,500–5,000 cells were seeded in 6-well plates and allowed to grow for 14 days. Colonies were stained with a crystal violet solution and then imaged. Colonies were analyzed by ImageJ (NIH) using the 3D Objects Counter with the minimum size set to 5.

### Wound healing assay

In total, 1 × 10^6^ cells were seeded in a 6-well plate and allowed to grow to confluence, approximately 24 hours. Media were removed from the wells and a p10 pipette tip was used to generate a cross through the well. Images were acquired at 0, 24, and 48 hours, using the cross section to ensure consistency in image acquisition. Wound closure was determined by assessing the area of the wound via a custom Cell Profiler pipeline. Wound area at 24 or 48 hours was divided by the wound area at 0 hours to determine relative wound closure.

### Spheroid invasion assay

Spheroids were created by seeding 300 cells in an ultra–low attachment round-bottom 96-well plate with 1 mg/mL fibronectin (Corning). Spheroids were allowed to form over 24 hours and then moved onto a flat-bottom 96-well plate coated in 0.5 mg/mL Matrigel in standard culture media. Images of the spheroids were acquired at 0 hours, 24 hours, and 4 days. Images were uploaded to ImageJ, and the area of the spheroid was determined using the Analyze>Measure tool. The invasion area was determined by dividing the area of the spheroid at the later time point (24 hours or 4 days) to the area of the spheroid at 0 hours.

### Xenograft models

Animal protocols were compliant with Washington University School of Medicine Animal Studies Committee regulations (IACUC protocol no. 21-0230). Eight-week-old immunodeficient (NOD/SCID) female mice were procured from The Jackson Laboratory and were housed in a sterile barrier facility prior to the start of the experiments. OVCAR cell lines were suspended in 50% Matrigel (Corning) in sterile PBS (Corning) and injected into the ventral left side of the peritoneal cavity. All mice were engrafted with 1 × 10^7^ cells in 100 μL total volume. Weight, abdomen width, and tumor burden were assessed weekly.

#### BLI.

Tumor growth and distribution were assessed by BLI with a Spectral Imaging AMI. Mice were injected i.p. with D-luciferin (150 μg/g in PBS, Goldbio, eLUCNA) and imaged 10 minutes later with the AMI (1s exposure, binning 8, f-stop 1). Total photon flux (photons per second) was quantified using the Spectral Imaging Aura software (Spectral Instruments). The region of interest was consistent throughout the experiment and encompassed the entire field of view for each mouse.

#### Tumor assessment and histopathology.

At the time of death or 165 days after i.p. tumor injection, total tumor burden was assessed through macroscopic dissection. Distant metastatic tumor nodules to the lungs were counted. Histopathology for the lung tissue was performed by Washington University School of Medicine Anatomic and Molecular Pathology Core labs. H&E staining, WT1, and TP53 IHC were used to determine the presence of ovarian tumor within the lung tissue. All histopathology was assessed and imaged in collaboration with an anatomic pathologist with expertise in gynecologic malignancies.

### RNA extraction and RNA-Seq

RNA was harvested from cell pellets using the Monarch Total RNA Miniprep Kit (New England Biolabs). Library preparation and sequencing was performed at Washington University School of Medicine GTAC.

#### Library preparation.

Total RNA integrity was determined using Agilent Bioanalyzer. Ribosomal RNA was removed by an RNase-H method using RiboErase kits (Roche). mRNA was reverse transcribed to yield cDNA using SuperScript III RT enzyme (Invitrogen, per manufacturer instructions) and random hexamers. A second strand reaction was performed to yield double-stranded cDNA. cDNA was blunt-ended and 3′ A-tailed followed by ligation of Illumina sequencing adapters to the ends. Ligated fragments were then amplified for 12–15 cycles using primers incorporating unique dual index tags. Fragments were sequenced on an Illumina NovaSeq using paired end reads extending 150 bases.

#### Differential gene expression analysis.

RNA-Seq reads were aligned to human reference genome (GRCh38.p14) with STAR version 2.5.1a. All gene counts were then imported into the R/Bioconductor package EdgeR, and TMM normalization size factors were calculated to adjust for samples for differences in library size. Technical triplicates for each cell line were assessed by RNA-Seq, and the average expression for each gene identified was calculated. DEGs were identified using DESeq2. DEGs were filtered using an adjusted *P* < 0.01 and fold change of > 1 or < –1 for OVCAR3-A3A V1–V3 and > 0.5 or < –0.5 for OVCAR4-A3A V1–V3. For WUSTL Met patient cohorts, DEGs were identified for GSEA using Limma to compare average gene expression between WUSTL Met APOBEC3 High and WUSTL Met APOBEC3 Low patient datasets. For EMT Trajectory Score analysis, DEGs were identified for each patient in the WUSTL Met APOBEC3 High dataset by determining fold change for each gene relative to the average expression of the gene in the WUSTL Met APOBEC3 Low patient dataset (fold change = [WUSTL Met APOBEC3 High expression-WUSTL Met APOBEC3 Low expression average]/WUSTL Met APOBEC3 Low expression average).

#### GSEA.

GSEA was performed to identify MSigDB Hallmark gene sets (95). The expression value for each gene identified in Hallmark epithelial-mesenchymal transition gene set was assessed and a heatmap was generated using Phantasus as described previously (96).

#### EMT trajectory score.

The EMT trajectory score was derived from expression data from a curated list of genes associated with epithelial, hEMT, or mesenchymal cancer cell phenotypes, as previously described (38). The differential expression values for each gene attributed to the given phenotype was averaged, and for hEMT and mesenchymal gene scores, the average expression value for the epithelial-associated genes was subtracted from the corresponding average expression value for hEMT or mesenchymal genes as previously described (38).

### ELISA

Cells were grown for 72 hours, after which culture media were collected and immediately assayed for IL-6 levels (Human IL-6 Quantikine Elisa, R&D Systems). Optical density at 540 nm was determined using a SPECTROstar Omega (BMG Labtech). A standard curve was generated by using a serial dilution of the IL-6 standard in duplicate. Values were averaged and plotted, and a best-fit line was generated in Excel. The concentration of IL-6 in the culture media was determined using the linear trendline equation.

### IL-6 blockade

Cells were treated with 50 μM/mL tocilizumab (IL-6 receptor inhibitor). For migration assays, treatment was initiated concurrent with a wound being created. For invasion assays, treatment was added to the media upon seeding of spheroids into ultra–low attachment 96-well round-bottom plate. At 24 hours, spheroids were moved to wells containing 0.5 mg/mL Matrigel with tocilizumab.

### IL6-correlated gene score

The *IL6-*correlated gene score was derived from expression data from a curated list of genes coexpressed with *IL6* that were correlated with specific biological functions (inflammation, metabolism, cell cycle control/apoptosis, motility/adhesion, and proliferation) (67). From triplicate RNA-Seq data, the expression values for each gene associated with a specific cell function were totaled. The sum of the expression values for genes associated with each cell function for OVCAR-A3A cell lines was subtracted from the sum of the expression values from the corresponding NT cell lines to generate a final *IL6-*correlated gene score.

### Statistics

All statistical tests were performed in R or GraphPad (Prism). Biological and/or technical triplicate tests were used to ensure robustness and reproducibility of data. *P* values were generated using paired and unpaired 2-tailed *t* tests, Fisher’s test, or 1-way ANOVA. Data are shown as mean ± SD.

### Study approval

Two cohorts of human tumor genomes were analyzed. All samples, including primary and metastatic lesions, were collected prior to treatment. Collection of sample and associated data were approved by IRBs for both cohorts: study no. 201309075 was approved by the WUSTL IRB; the DFCI/Penn study was approved by the IRB at Brigham and Women’s Hospital and the Johns Hopkins Hospital, and all patients gave informed consent before inclusion. WUSTL cohort patients were included if they had FIGO stage III–IV ovarian cancer of serous histology. Animal protocols were compliant with Washington University School of Medicine Animal Studies Committee regulations (IACUC protocol no. 21-0230).

### Data availability

Variant calling from genome sequencing of patient tumors in WUSTL cohort, RNA-Seq and variant calling from genome sequencing of OVCAR cell lines, and all code used for the analysis are available at https://doi.org/10.5281/zenodo.12571514 RNA-Seq files from patient tumors in the WUSTL cohort are deposited in the NCBI GEO database under accession no. GSE218989. WES data from patients in the WUSTL cohort are available from the NIH Sequence Read Archive under the accession no. SRX20007159. All supporting data values for main manuscript and supplemental materials are available in Supporting Data Values file.

## Author contributions

JMD, BRH, RAD, DF, DL, and LHE performed experiments and analyzed and interpreted data. LS performed histopathological imaging and interpretation. TT and RD performed informatic experiments and statistical tests. JMD, AMG, KF, JJB, MMM, and RD conceptualized and designed experiments. JMD and AMG wrote the manuscript, with editing from all authors.

## Supplementary Material

Supplemental data

Unedited blot and gel images

Supplemental table 1

Supplemental table 2

Supplemental table 3

Supporting data values

## Version 1 03/10/2025

Electronic publication
