# Supplementary figures and images for: APOBEC3A drives ovarian cancer metastasis by altering epithelial-mesenchymal transition
Source: JCI Insight. 2025 Mar 10;10(5):e186409. doi: 10.1172/jci.insight.186409 (PMC11949045; doi:10.1172/jci.insight.186409)

# OVCAR3 A3A

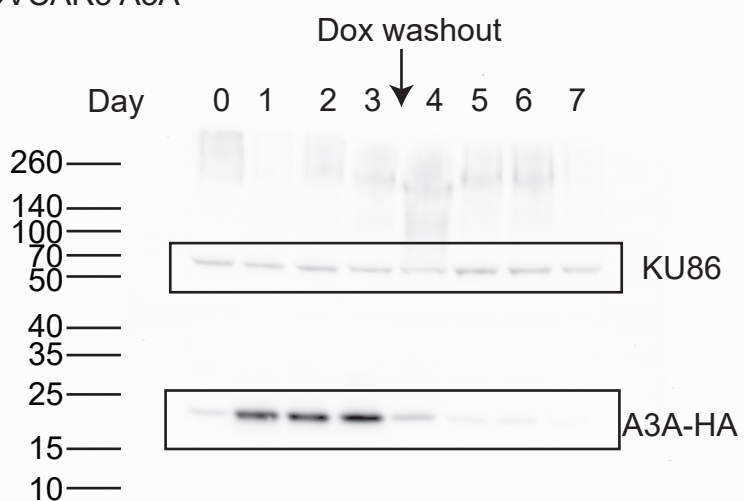

# OVCAR4 A3A

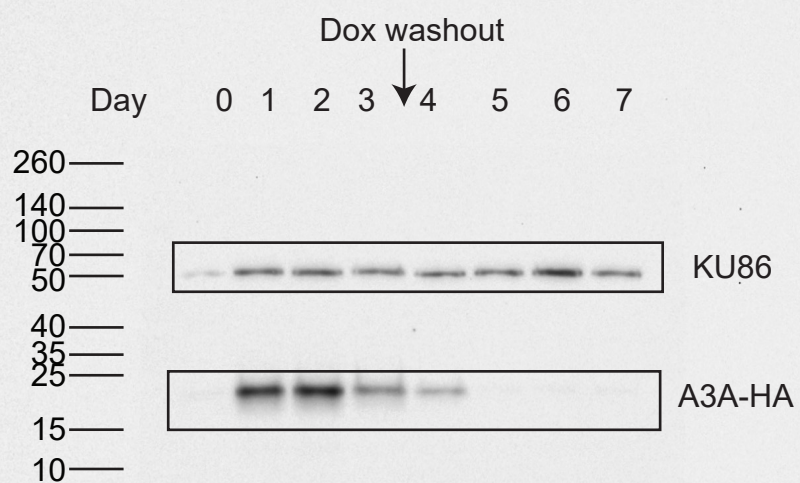

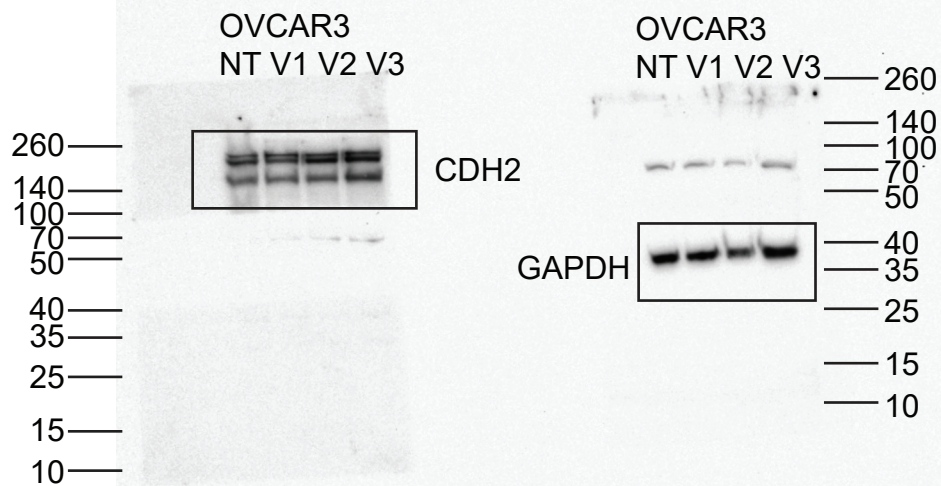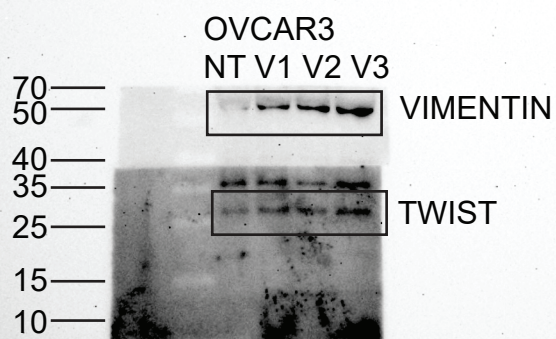

Supplement: Unedited blot and gel images [file jciinsight-10-186409-s162.pdf]
